# μCT imaging of a multi-organ vascular fingerprint in rats

**DOI:** 10.1371/journal.pone.0308601

**Published:** 2024-10-14

**Authors:** Hanna Napieczyńska, Sarah M. Kedziora, Nadine Haase, Dominik N. Müller, Arnd Heuser, Ralf Dechend, Kristin Kräker

**Affiliations:** 1 Max‐Delbrück‐Center for Molecular Medicine in the Helmholtz Association (MDC), Berlin, Germany; 2 Corporate Member of Freie Universität Berlin, Humboldt Universität zu Berlin, and Berlin Institute of Health, Charité–Universitätsmedizin Berlin, Berlin, Germany; 3 Experimental and Clinical Research Center–A Joint Cooperation between the Max Delbrück Center for Molecular Medicine and the Charité–Universitätsmedizin Berlin, Berlin, Germany; 4 DZHK (German Center for Cardiovascular Research), Partner Site Berlin, Berlin, Germany; 5 Department of Cardiology and Nephrology, HELIOS Clinic, Berlin, Germany; Icahn School of Medicine at Mount Sinai Department of Pharmacological Sciences, UNITED STATES OF AMERICA

## Abstract

The importance of microvascular imaging in diagnosis and therapeutic targeting of various diseases is increasingly recognized. The new approach emphasizes the need for holistic studies to understand the inter-organ vascular cross-talk. Here, we report on the development of a novel perfusion protocol which consistently delivers a micro-computed tomography contrast agent to micro-vessels of multiple organs in a single experimental animal. We describe the achieved repeatability of the perfusions, as well as the image analysis steps developed individually for each organ type. We also optimize image acquisition by investigating the compromise between shortening of the scanning time and preservation of the highest possible spatial resolution. Taking together, with the multi-organ perfusion, optimized image acquisition, and the conceived image analysis steps, we provide a comprehensive and reliable experimental protocol for studying vascular morphology and pathology in multi-organ diseases.

## Introduction

Microvascular alterations are the main pathophysiological factor in a wide range of diseases and represent targets for potential novel treatments. However, diagnostic modalities to visualize or quantify microvascular changes in various organs are limited. Today only proteinuria and retinal vasculopathies can be considered as potential windows for the presence of microvascular alterations in humans [1–5]. Moreover, suitable animal models are essential to study the cross-talk of organs and their complex interactions within an organism.

One approach to these challenges is using micro-computed tomography (μCT) for vascular imaging in preclinical studies. The technique relies on perfusing an experimental animal with a contrast agent and subsequently extracting the organs for high resolution imaging. This provides detailed 3D models of the vascular networks of entire organs, which can be analyzed quantitatively. However, the perfusion method is usually optimized for a single organ type. Until now, μCT imaging of microvascular structures has been shown for heart [6], brain [7] kidney [8–10], or the uterus during pregnancy in mice [11]. Unfortunately, focusing on a specific organ type does not allow investigating possible interactions between the vascular systems of multiple organs within the same subject. Although perfusion protocols utilizing a more holistic approach have been proposed, they enabled visualization of only relatively large blood vessels, such as coronary arteries in the heart or blood vessels larger than 50 μm in the diameter in the kidney [12, 13].

Therefore, the main goal of this study was to develop a novel multi-organ perfusion protocol which enables μCT imaging of blood micro-vessels in diverse body organs. To further advance the method, we have also developed workflows for a quantitative image analysis of every organ type. Finally, we focused on evaluating different image acquisition approaches to balance the highest possible spatial resolution in the images with the affordable acquisition time. Thus, we provide a complete description of the experimental protocol for multi-organ micro-vessels μCT and present intriguing findings observed with the established procedures.

## Methods

### Animals

Four male Sprague-Dawley rats (ID #19061–64) were used to establish the perfusion and image analysis protocols. They were housed in a temperature-controlled environment of 22±2°C, 55±15% humidity and a 12:12-hour light/dark cycle, with *ad libitum* access to food (Sniff V1324-300) and water. The rats were siblings and sacrificed at the age of 18 weeks.

For evaluating the impact of the image acquisition settings on the image quantification, organs of four female Sprague-Dawley rats were used. These females carried the human angiotensinogen gene [TGR(hAogen)L1623], but did not express the pathological phenotype. They were housed under the same conditions and diet as the male rats.

The animal experiments were performed under predefined stopping criteria according to the European law for animal protection. The studies were approved by the local authorities (State Office of Health and Social Affairs Berlin, approval number Y9004-18).

### Multi-Organ perfusion

**Fig 1:**
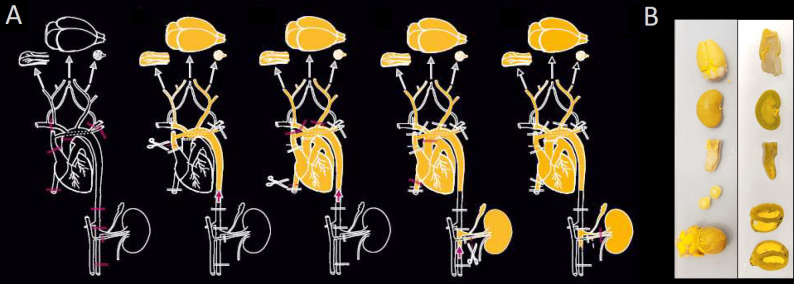
**A.** The multi-organ perfusion protocol requires several occlusions, incisions, and cannulations. The contrast agent is first perfused into the brain, eyes, and tongue (left), then into the heart (middle), and finally into the kidney (right). **B.** The uniform delivery of the contrast agent into the vascular network is visible in the harvested organs (left: whole organ, right: long-axis section).

The animals were overdosed with isoflurane (4%, CP-Pharma, Germany) and a Y-shaped laparotomy was performed. The subsequent perfusion procedure required multiple clamps, incisions, and cannulations, as shown in Fig 1A.

In the first step, the *superior* and *inferior vena cava*, left and right subclavian arteries and brachiocephalic vein were closed with suture (1.5, 4/0, SMI, Belgium). A cannula (18G, B. Braun Melsungen, Germany) was then inserted into the aorta proximal to the renal arteries. Blood was flushed out through an incision in the *superior* and *inferior vena cava* with 1x phosphate-buffered saline (Gibco 10xPBS pH7.4, Fisher Scientific, Germany) supplemented with heparin (25IU/5mL, B. Braun Melsungen, Germany). Next, the ascending aorta was temporarily clamped proximal to the heart. The freshly mixed Microfil solution (MV-122, Flow Tech Inc., United States, in the ratio 1 mL diluent: 1 mL pigmented solution to 0.1 mL hardener) was infused at a constant rate of 14 mL/min through a cannula using a peristaltic perfusion pump (ISM834C, ISMATEC, Germany). The *superior vena cava* was additionally occluded cranially to the incision as soon as Microfil was leaking out. The brachiocephalic and left common carotid arteries were quickly closed in a proximal sector to prevent Microfil leakage during hardening. In the second step, the temporary clamp was removed from the ascending aorta to channel Microfil into the heart. The *inferior vena cava* and ascending aorta were closed proximally to the heart once Microfil appeared consistently at the *vena cava* incision and the coronary arteries were observably filled. In the final step, a second cannula (18G, B. Braun Melsungen, Germany) was inserted into the infrarenal aorta. Blood was flushed out from the kidney through a renal vein incision with 1x PBS/heparin solution. The freshly prepared Microfil solution was applied in the same ratio as described above. The left renal vein and artery were closed when Microfil continuously appeared through the incision. All the procedures, including animal and vessel preparations, as well as perfusions, lasted up to 1 hour per rat. After the Microfil hardening for 3 hours at room temperature, the hearts, kidneys, brains, tongues, and eyes were harvested and stored in 4% PFA (pH 7.4, Morphisto, Germany) at 4°C until μCT was performed, no longer than 7 days after organ harvesting. The brains were additionally stored in 8% formic acid (Sigma Aldrich, Germany) at room temperature for 48 hours to decalcify the skull and facilitate removal of the brain.

Due to the unavailability of Microfil for the second part of the study, the female rats were perfused with a different contrast agent, Vascupaint (MDL-121Y, MediLumine, Canada). The perfusion procedure remained the same.

### μCT Imaging for evaluation of the perfusion protocol

**Fig 2:**
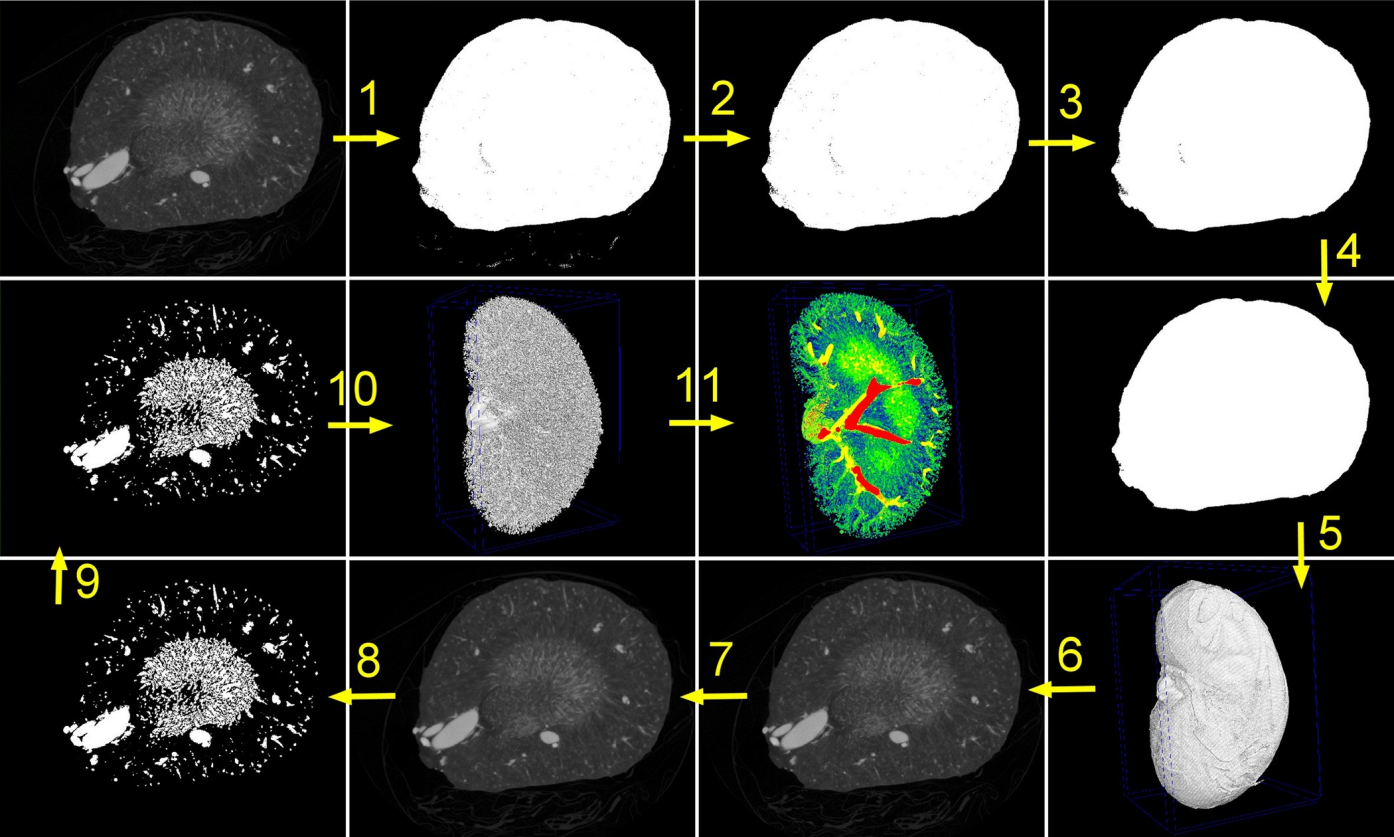
Steps of the image analysis protocol. 1. Thresholding for total volume. 2. Sweeping all except for the largest object. 3. Removing black speckles. 4. Closing. 5. Computing total organ volume. 6. Reloading original image. 7. Filtering. 8. Thresholding for vessels. 9. Removing white speckles and closing. 10. Computing vascular volume. 11. Computing vessel diameters.

The organs were wrapped in a piece of foil (with an additional piece of wet tissue beneath for eye samples, due to their particular susceptibility to loss of water and possible shrinkage) and imaged with SkyScan 1276 (Bruker, Belgium), using the vendor’s software for image acquisition (v.1.4.0.0). The step and shoot mode with 360° acquisition were applied. The X-ray source current was 200 μA and other parameters were adjusted individually for every organ type, as listed in S1 Table. An adequate flat field correction was always applied. Image reconstruction was performed with NRecon (v.1.7.3.1, Bruker, Belgium) applying the beam-hardening correction of 6% (eye), 16% (brain), 21% (kidney and tongue), or 42% (heart), as well as the ring-artefact correction of 5 (eye, heart), 3 (brain), 2 (kidney), or 4 (tongue). Image analysis was performed with CTAn (v.1.20.3.0, Bruker, Belgium) by applying a sequence of image analysis steps, as shown in Fig 2. This analysis led to calculating the total volume of the organ, the volume of the vascular tree and the distribution of the vessel diameters. The exact parameters used for the image analysis of each organ type are presented in S2 Table.

The logic behind selecting the specific parameters was the following: The threshold values for binarization for total volume were chosen to include the complete tissue, allowing for possible pores inside the volume, if unavoidable. The “Removing black spackles” and “Closing” procedures were then applied to remove those pores. Filtering was performed after reloading of the image to facilitate the subsequent binarization of the vascular network. The thresholds used for this binarization were chosen to preserve as much physiological shape of the vascular structures as possible. In the case of the heart images, a compromise had to be found to account for some loss of small objects and artificial overestimation of the large ones. The “removing white spackles” procedure was then applied to remove tiny elements that remained in the image after the binarization but originated from the noise, rather than from the true signal.

The consistency of the perfusions among animals was evaluated qualitatively as well as quantitatively. The former was based on 3D rendering of the obtained images, which was performed with CTVox (v.3.3.1.0, Bruker, Belgium), while the distributions of the vessel diameters were used for the latter. The summed volume of the vessels with the diameter of up to 0.4 mm was considered the total vascular volume of the organ (100%). The volumes of the vessels within given diameter ranges are expressed as fractions of the total volume (% total). The group standard deviations were used as a measure of data spread.

### Optimization of μCT acquisition

The μCT hardware and software described in the previous section were used, but every sample was imaged three times. Each time, a different current of the X-ray tube was applied to vary the X-ray focal spot, influencing the effective spatial resolution. The exposure time was adjusted accordingly to match 30% of the minimal transmission of the X-rays through the sample. With this, the following acquisition protocols were used:

1. *highest resolution*: power of 4 W–the smallest focal spot,
2. *fastest scanning*: power of 15–20 W–the largest focal spot,
3. *intermediate parameters*: power of 8 W–intermediate focal spot.

The remaining acquisition parameters were the same as described in the previous paragraph, with an exception for the pixel size, which this time was set at 5 μm for all the organs (S3 Table).

Image reconstruction and analysis were performed as described above. The values of the thresholding steps in the image analysis were adjusted for the images obtained with approaches 1) and 3). The distributions of the vessel diameters computed from the images acquired with the three protocols were compared, using the output of “highest resolution” protocol as a reference.

In addition, a MicroCT Bar Pattern NANO Phantom (QRM, Germany) was also scanned three times, applying acquisition parameters of the eye samples. This was done to evaluate the highest possible spatial resolution that could be achieved with the selected settings.

### Immunostaining

For hematoxylin and eosin (H&E) staining, the deparaffinized and rehydrated sections (3 μm thick) were incubated with hematoxylin (Roth, Germany) for 8 min and washed for 15 min in tap water. Afterwards, the sections were placed in eosin (0.5%, Roth, Germany) for 3 min, dehydrated using an ascending ethanol series (96%, 2x 100%) and 2 x 5 min xylene (Roth, Germany). Finally, the sections were covered with Eukitt (Sigma Aldrich, Germany). Wheat germ agglutinin (WGA; Vector Laboratories, USA), immune cells (CD68; MCA341R, Bio-Rad Laboratories, USA), fibronectin (Fn; Ab23751, Abcam, UK) and collagen type 1 (Col1; 1310–01, Southern Biotech, USA) were stained via protocol presented in S4 Table.

## Results

### Qualitative evaluation of the perfusion protocol

The established protocol allowed an efficient and homogeneous delivery of the contrast agent to multiple organs within a single experimental animal. This was achieved in three major steps: the eyes, brain and tongue were perfused first via the carotid arteries, while the inflow to the heart was briefly clamped off at the descending aorta. In the second step, the heart was perfused. Finally, for perfusion of the kidney, a second cannula was placed in the infrarenal aorta (Fig 1A). With this multi-stage procedure, the vascular networks became easily visible in all the extracted organs (Fig 1B).

**Fig 3:**
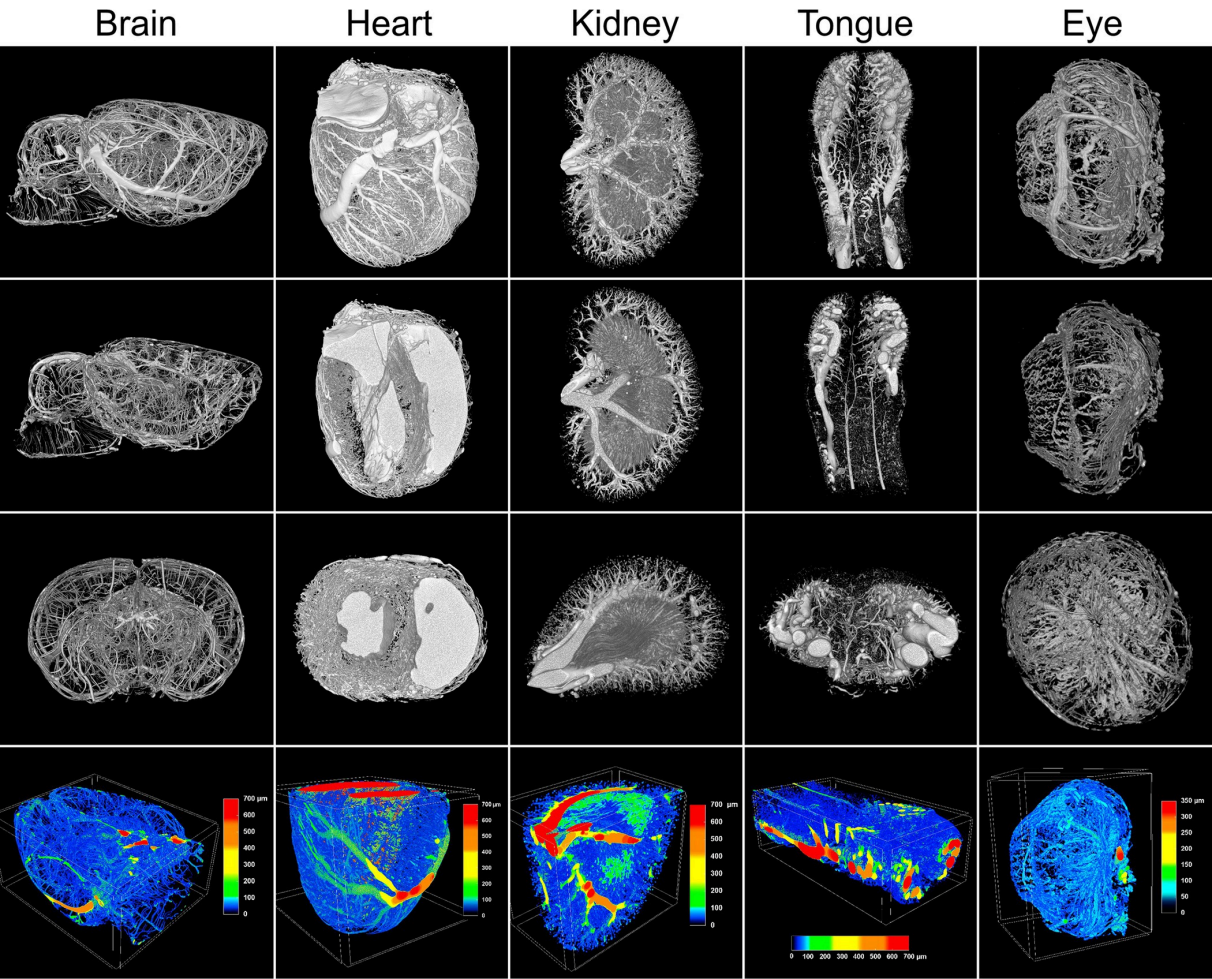
μCT images of vascular networks in different organs of one rat. Sagittal and coronal sections are shown in the second and third panel from the top, respectively. The color-coded diameters of the blood vessels are depicted in the bottom panel.

The efficiency of the perfusion was also clearly visible in the μCT images. An example showing all the organs of one rat, including visualizations of the blood vessel sizes computed in the last step of the image analysis, is shown in Fig 3. 3D animations of these images are available in S1–S5 Videos and analogical images of the remaining rats are shown in S1 Fig. Based on the qualitative assessment of the images, brains, kidneys, and tongues were perfused very consistently in all the animals, while a limited variability in the extent of chamber filling occurred in the hearts.

### Quantitative evaluation of the perfusion protocol

**Fig 4:**
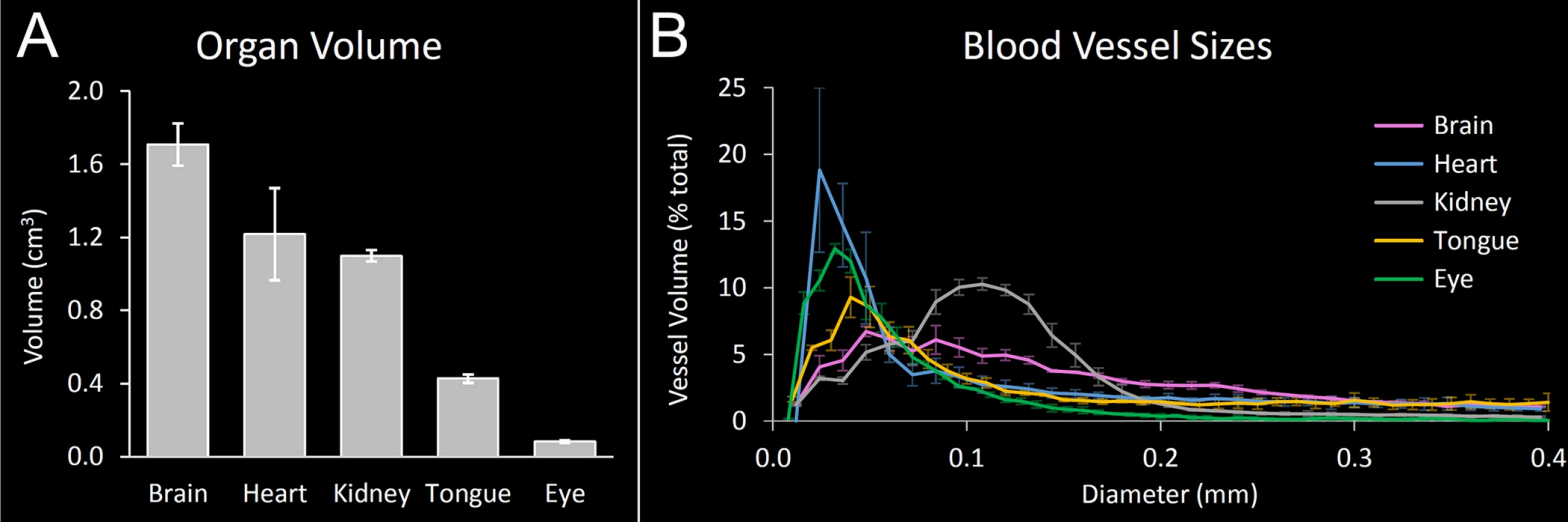
Organ and blood vessels sizes computed from μCT images. **A.** Whole organ volumes. **B.** Perfused vessel volume per vascular diameter defines a unique vascular fingerprint of rat organs. The graphs show means ± stds (n = 4).

To verify the consistency of the perfusions in a quantitative manner, volumes of the entire organs as well as the diameters of the blood vessels were computed in the μCT images, as described in the Methods section. Most total sample volumes were very similar in all four rats, as could be expected for the animals of the same age and sex. They were: 1.10 ± 0.03 cm^3^ for the kidney, 0.43 ± 0.02 cm^3^ for the tongue, and 0.08 ± 0.01 cm^3^ for the eyes (mean ± sd). The volumes of the brains varied minimally (1.71 ± 0.12 cm^3^), while the variability in the volume among the hearts was larger, 1.22 ± 0.25 cm^3^ (Fig 4A).

Independently of the total organ volumes, distributions of the blood vessel diameters were computed and averaged per organ type. The standard deviations of the means were used as a measure of data spread and the results are shown in Fig 4B. For the entire evaluated spectrum of the vessel diameters, *i*.*e*. up to 400 μm, the largest standard deviations were: 1.6% for the brains (at 50 μm), 6.2% for the hearts (at 20 μm), 0.9% for the kidneys (at 80 μm), 1.5% for the tongues (at 50 μm), and 1.1% in case of the eyes (also at 50 μm). These results demonstrate a very low variability of the measured vessel sizes among the four rats, particularly in the kidneys and eyes, followed by tongues and brains. The standard deviation was slightly larger in the hearts only, reflecting the slightly lower consistency of the perfusions in this organ, as previously noticed during the visual inspection of the images.

### Vascular fingerprint

Interestingly, when we compared the vessel size distributions of different organs, a characteristic pattern could be identified for each organ type (Fig 4B). The peak volume of the cardiac vessels was around 20 μm diameter (18.8 ± 6.2%) and more than half of the vascular volume was contained in the vessels with the diameter smaller than 80 μm. In turn, the distribution of the cerebral vessels was characterized by a flatter shape, with the largest volume at the diameter of 50 μm (6.7 ± 1.6%). Half of the cerebral vessel volume was localized in the vessels of up to 130 μm in diameter. Renal vessels showed the highest volume at the diameter of 110 μm (10.3 ± 0.4%) and covered half of their volume under this peak. The blood vessels of the eye contained the largest volume at the diameter of 30 μm (12.9 ± 0.4%) and half of their vascular volume was in the vessels of 50 μm in diameter or smaller. Finally, the lingual vessels presented the highest volume at the diameter of 40 μm (9.3 ± 1.5%) and half of the vascular volume in the tongue was localized in the vessels not larger than 100 μm. We called these characteristic distribution patterns „vascular fingerprints”of the organs.

### Immunostaining

**Fig 5:**
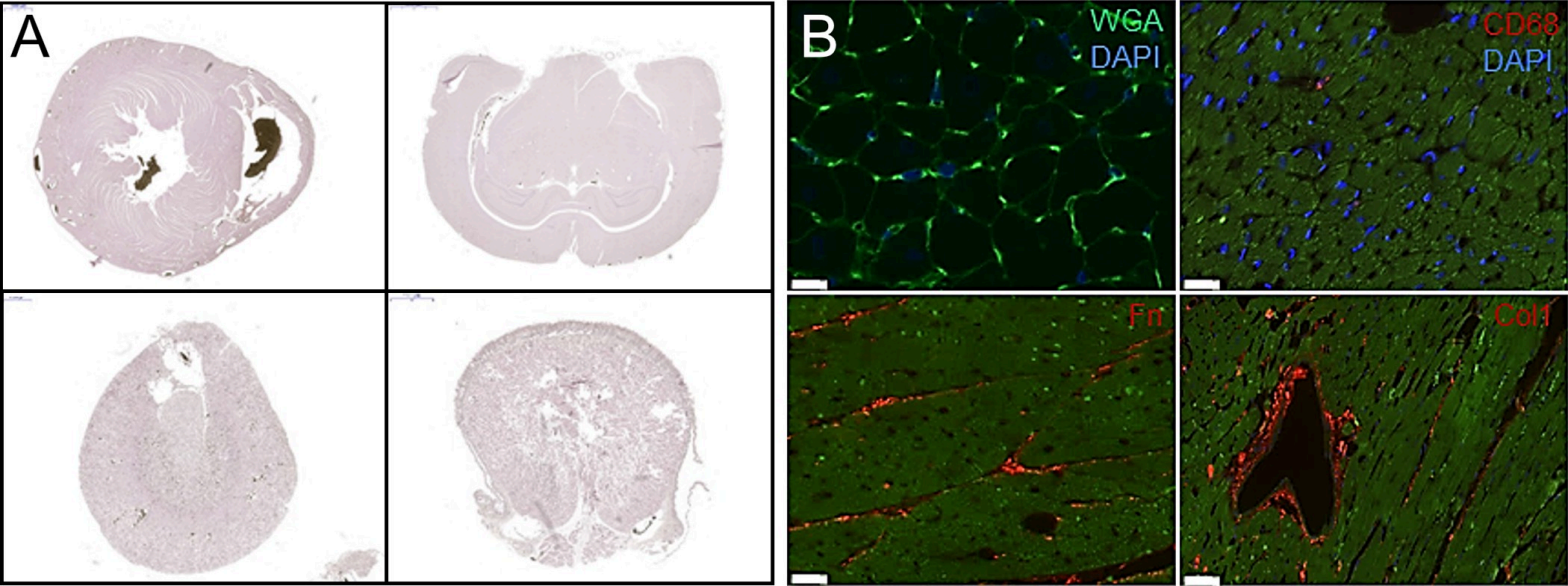
Immunostaining of the tissue was performed after μCT imaging. **A**. Examples of H&E staining in heart (top left), brain (top right), kidney (bottom left) and tongue (bottom right). **B.** Example slices of immunohistochemical staining against wheat germ agglutinin (WGA, top left, scale: 10 μm), immune cells (CD68, top right, scale: 20 μm), fibronectin (Fn, bottom left, scale: 20 μm), and collagen type 1(Col1, bottom right, scale: 50 μm).

After perfusion and μCT imaging, immunostaining of all organs was conducted with adjusted protocols as described in the Methods section. In addition to the H&E staining of the heart, brain, kidney, and tongue tissues (Fig 5A), fluorescence immunostaining was performed as well (Fig 5B). It allowed visualization of the size of the cardiomyocytes with wheat germ agglutinin (WGA) staining, as well as the immune cells with CD68 antibodies. Interstitial (fibronectin, Fn) and perivascular fibrosis (collagen type 1, Col1) staining could be also carried out for exploring potential relations between microvascular alterations and structural remodeling.

### Optimization of image acquisition

In the second part of this study, we focused on optimizing the image acquisition parameters, with the goal of maximizing spatial resolution at the possibly short acquisition time. For this purpose, we first scanned a resolution phantom and then the rat organs. In both cases three different sets of power settings of the X-ray source were applied.

**Fig 6:**
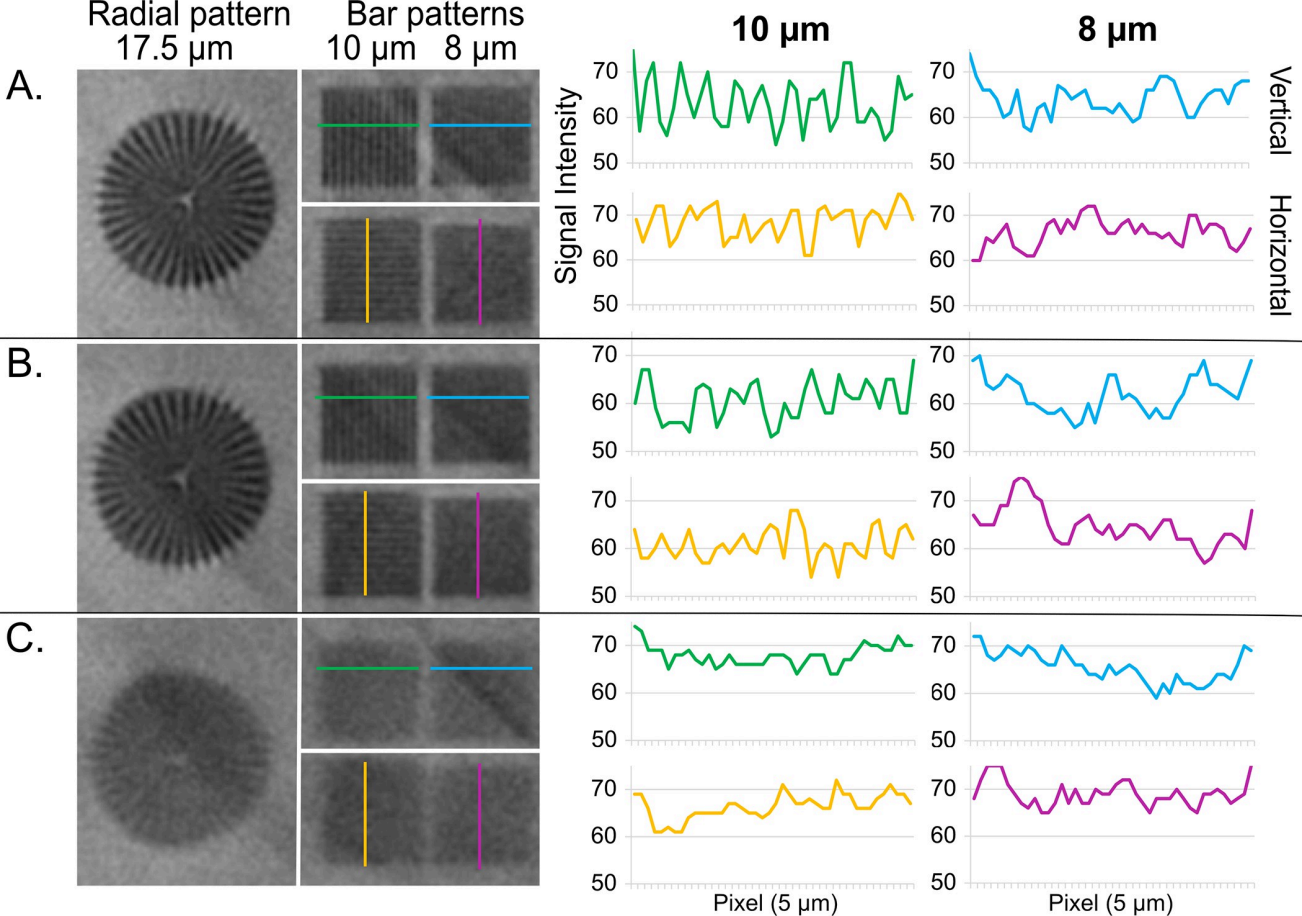
μCT images of a resolution phantom reveal the smallest dimensions that could be resolved using three image acquisition protocols: **A.** “Highest resolution” protocol, **B.** “Intermediate Settings”, and **C.** “Fastest scanning”. 10 μm bars can be resolved in A and (to a slightly lower extent) in B.

With the lowest power of 4 W („highest resolution”settings), 10 μm bar pattern could be clearly resolved in the phantom image, while resolving the 8 μm bars was already compromised (Fig 6A). With the frame averaging of 3, the scanning time of a single field of view (FOV) was 5h 45min. Compared to this, imaging at 15 W was much faster and a single FOV required 2h 25min only. However, neither 8 μm, nor even 10 μm, bars could be resolved (Fig 6C). The in-between mode („intermediate parameters“, X-ray source power of 8 W) saved substantial scanning time, with the acquisition lasting 3h 20min per FOV. The 10 μm bars could be still resolved, although a little less clearly than in the image acquired with the „highest resolution”settings (Fig 6B).

**Fig 7:**
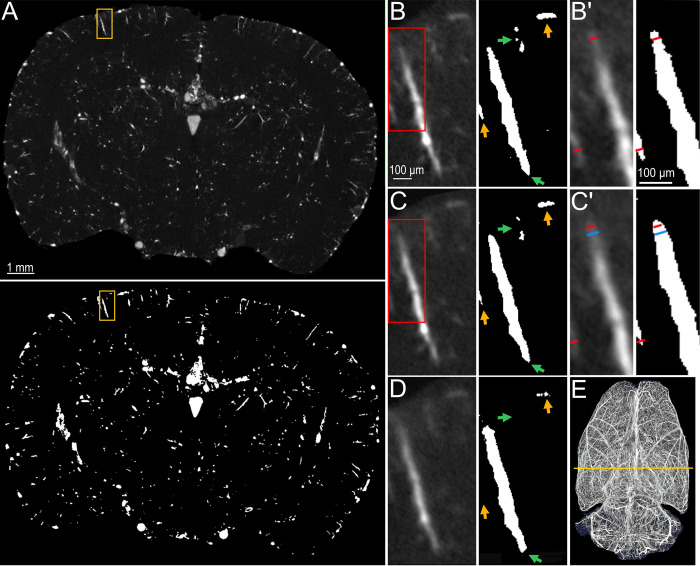
**A.** One slice through the μCT image of a rat brain acquired with the “Highest Resolution” protocol (upper) and the same slice after image segmentation (lower). The yellow rectangles indicate areas magnified in the next panels. **B-D.** Magnified fragments of the original images (left) and outputs of the image segmentation (right) acquired with the “Highest Resolution” (B), “Intermediate Settings” (C) and “Fastest Scanning” (D) protocols. The arrows in the images after segmentation show loss of details gradually occurring as spatial resolution drops. The red rectangles in B and C indicate sections magnified in B’ and C’, correspondingly. **B’-C’.** A magnified view on a fragment of a blood vessel with its dimensions measured in the original images (left) and in the output images (right). The red lines represent 20 μm and the blue lines represent 30 μm. **E.** Maximum intensity projection of the whole brain with the line indicating position of the analyzed image slice.

In the imaging of the rat organs, the „highest resolution”protocol provided very detailed visualizations of the whole organ vascular networks. An example of a brain image is shown in Fig 7 and another example, presenting the dense vascular network of the tongue, as well as an interesting visualization of the tongue surface with its papillae, is presented in S2 Fig. In these images, blood vessels of 20–25 μm width could be clearly seen and measured and this dimension was mostly preserved after image segmentation (Fig 7B–7B‘). Some smaller blood vessels (15–19 μm wide) could be also found, although the signal intensity of these tiny objects was usually lower, compromising the accuracy of image analysis. The spatial resolution in the images acquired with the „intermediate settings”protocol was still very high, although some smallest elements could be lost over image segmentation (Fig 7C–7C‘). The images obtained with the „fastest scanning”protocol were characterized by the visibly lowest spatial resolution, which was also reflected in the binarized images (Fig 7D).

**Fig 8:**
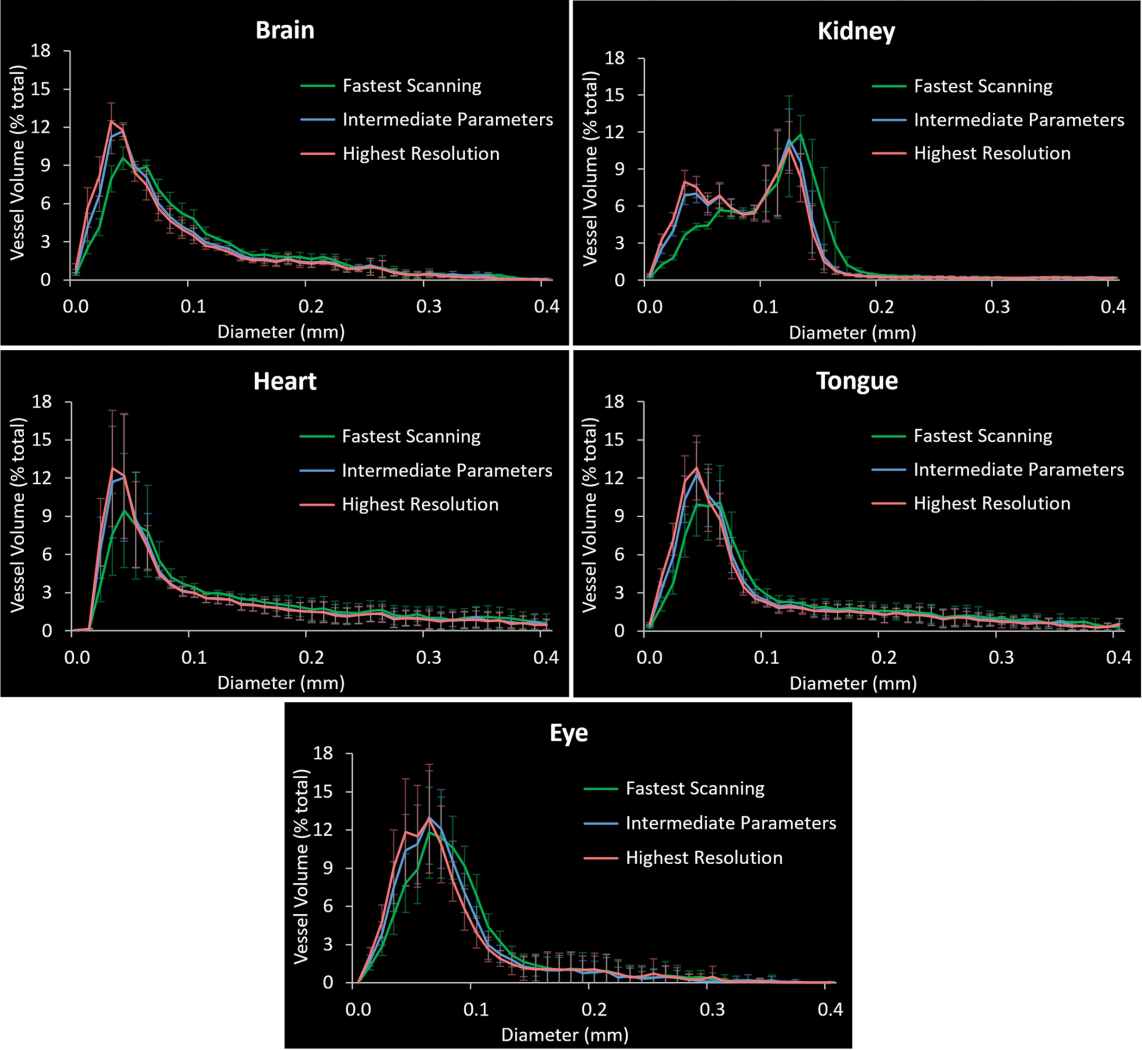
Comparison of the distributions of the blood vessel diameters computed from μCT images acquired with three different protocols. In all the organs, the volume of the blood vessels with the small diameters was clearly underestimated in the images obtained with the “Fastest Scanning” protocol as compared to the “Highest Resolution” one, which served as a reference. In turn, the results obtained with the “Intermediate Parameters” protocol only slightly deviated from the reference values. Each data point represents a mean ± sd (n = 4, except for the data of the eyes acquired with the Highest Resolution protocol where n = 3).

Finally, to examine the differences in the images acquired with the three protocols in a quantitative manner, the distributions of the blood vessel diameters were compared. As expected, the portion of the vessels with the smallest diameters was substantially underestimated in the images acquired with the „fast scanning”settings compared to the „highest resolution”protocol which served as a reference. This was true for all the tested organ types and with the underestimation present in the diameters of up to 50–60 μm (Fig 8). In turn, the volume of the vessels in the diameter ranges of 80–120 μm (brain, tongue, and eye) and 120–150 μm (kidney) was clearly overestimated.

In the most important comparison, the distributions computed from the images obtained with the „intermediate parameters”protocol were characterized by almost the same shape as the diameter distributions computed from the „highest resolution”images. The marginally lower volume was found for the vessels with the diameters of 30 and 40 μm only, demonstrating reliability of the intermediate power settings for quantitative μCT of blood vessels.

The total organ volumes were also computed from these images and the results are shown in S3 Fig. They demonstrate a good stability without tissue shrinkage of the samples over the period of image acquisitions.

## Discussion

To establish a reliable experimental pipeline for high-resolution μCT imaging of vascular networks in multiple organs, we have introduced a new perfusion protocol, individualized image analysis for each organ type and optimized image acquisition.

The main value and novelty of the perfusion protocol lie in the holistic attitude to the organism combined with the sequential approach to the procedure. This approach contrasts traditional whole-body perfusions performed in a single step via the apex of the left ventricle of the heart [12, 14]. Those protocols were developed for studying a vascular system of a single organ only [6, 7, 10] or delivery of the contrast agent was limited to the largest blood vessels, compromising analysis of micro-vessels in the entire 3D volume [12, 13]. With the developed method, we can now visualize microvascular structures in the primary target organs (heart, brain, kidney) as well as in organs important for translational approaches (eye and tongue). Thus, vascular networks of individual organs can be examined in extraordinary detail within a multi-organ context of the same subject.

Importantly, we demonstrate the reliability of the multi-organ perfusion protocol, as the distributions of the vessel diameters of a given organ type are very much alike throughout all the animals. The low standard deviations of the mean values quantitatively show high repeatability of this method. However, it should be indicated that achieving such results requires some technical skills and may be initially challenging for an untrained person. In our study, all perfusions were performed by one experimenter and obtaining similar results by a different person or research group would demonstrate reproducibility of the technique.

Furthermore, we have thoroughly investigated image analysis steps and optimized them individually for each organ type. This was a natural consequence of different morphology and sizes of the organs, implying slightly different settings for image acquisition. For example, the eyes, being smaller and containing less contrast agent than the brains, were imaged using 1 mm Al filter, while Al+Cu filter was used for the brains (S1 Fig). Another difference was due to a different distribution of blood vessels with different sizes throughout the organ volume. For instance, vessels of different sizes are distributed rather homogenously throughout most of the organ (and image) volume in case of the brain, while areas with a dominance of large or small vessels can be easier defined in the images of the hearts and kidneys. Consequently, global thresholding performed well in the segmentation step in the former, while adaptive thresholding provided a more accurate segmentation in the latter.

It is important to mention that the individual approach to the image analysis of different organ types was motivated by acknowledging the biological, morphological differences among these samples. The optimization of the image analysis workflows was carried out with a great care and with the goal of segmenting the vascular networks in each organ type as accurately as possible. Thanks to these efforts, we were able to observe a very intriguing result in the final step of the analysis–the “vascular fingerprint” of the organs (Fig 4B).

The term “vascular fingerprint” refers to the unique distribution of the vascular volume dependent on the vessel diameter. It appears to be characteristic for each organ type. The shape of this distribution can be described by the diameter, which is dominant in the given organ, and by the range of diameters from 0 to x μm, which covers half of the total vascular volume. These parameters were clearly different between the organ types we examined, although a higher animal number would be beneficial to further confirm this observation. We believe they could be used to quantitatively characterize microvascular beds in diverse pathological conditions, potentially serving as disease markers.

However, the idea of using these (or any other) parameters characterizing blood vessel sizes in a 3D image of an entire organ volume would still need to be validated using a different method. Up to now only a correlation between the blood vessel diameters measured in μCT images of the Microfil-perfused tissues and the diameters measured in microscopic images of the same tissues immunohistochemically stained against CD31 (a marker of endothelial cells) has been shown in 2D sections [15].

Nevertheless, a general description of the vascular sizes computed in 3D has been already used in one preclinical study. A reduction in the volume of smaller vessels (100–340 μm in diameter) was accompanied by an increased volume of larger vessels (340–740 μm in diameter) in the hearts of aged rats as compared to the young ones [6]. Another study found a change in the vascular volume fraction–although without measuring the vessel diameters–in different regions of the kidneys in rats subjected to a chronic bile duct ligation [16]. Since microvascular rarefaction or remodeling has been long described in animal models [17], and is known to occur in vascular pathologies in humans [18], we believe that a more detailed analysis of the vascular fingerprints will not only bring improvement in preclinical research but will also advance clinical studies. One known limitation of the method is the inability to distinguish arteries from veins, which could be relevant for some pathologies.

In humans, alterations in the distribution of microvascular sizes have already been recognized as a potential diagnostic feature in several diseases. Although only few microvascular beds are easily accessible, such as sublingual and retinal vessels, they could serve as a window to the body [1, 19]. For instance, it has been recently reported that the mean veins diameter in retinal structures was positively correlated with disease severity and negatively correlated with the time from symptoms onset in COVID-19 patients [20]. Retinal vessel analysis has been also shown as a useful tool in the risk assessment of metabolic diseases like diabetes [4], and it could be used to examine the effect of various medications or treatments [3]. The investigation of sublingual vessels as a potential non-invasive tool has been evaluated in patients suffering from sepsis [21, 22]. Thus, a closer focus on the vascular fingerprints may be extremely helpful in diagnosis and treatment of diverse diseases, potentially including also different forms of hypertension and vasculopathy. Currently available treatments often focus on disease symptoms or an isolated risk factor, but rarely target the micro-circulation itself. With our method, those vessel beds are also accessible in rodents and offer the reverse translation to investigate molecular mechanisms and vascular alterations at the micro-meter level.

In fact, the high spatial resolution achievable with μCT is a critical aspect of our approach. It determines the smallest size of the micro-vessels that can be reliably measured, which is particularly important if a global analysis method, handling the entire 3D image at once, is applied. For this reason, we have also evaluated three image acquisition protocols, which differ in the current of the X-ray tube. Changing this setting influences the spatial resolution by modulating the focal spot size of the source [23]. Lowering the current improves the resolution but simultaneously a longer exposure time must be used, significantly prolonging the total study duration. Hence, balancing the spatial resolution with the practicality of the experiment is an integral part of μCT research.

We used the power of 4W as the most optimal setting for vascular imaging in our system and we could resolve a 10 μm bar pattern in the image of a phantom (Fig 6). However, from the experimental perspective, the most important point was to examine what this means for biological samples perfused for vascular imaging. We have found that blood vessels with the diameter of 15–20 μm could be visualized and measured manually in the original images. However, this dimension would be lost over the image analysis since the analysis parameters were selected for handling the whole 3D image volume, and not only the smallest elements. With the sizes ranging from 15 to several hundred μm, even adaptive thresholding methods were not able to accurately segment the smallest and the largest objects in the image at the same time. Nevertheless, the diameters of 20–30 μm could be segmented mostly reliably (Fig 7) and the “intermediate settings” protocol provided almost the same quantitative results as the reference one (Fig 8). For comparison, in the above-mentioned studies, the blood vessels were measured to the limit of 80–120 μm and in one organ only [6]. Another report, in which human tissues (placentas) were of interest, analyzed blood vessels with the diameter down to 100 μm [24].

Beside the high spatial resolution, we have also demonstrated that the perfusions were repeatable, showing little variation in the measurements of a given organ type among several rats. This is an important factor enabling a proper quantitative data analysis of the experimental group, as opposed to measurements performed in individual animals only. However, one needs to be aware that too high a pressure of the contrast agent during perfusion could potentially result in vascular ruptures, which has been previously reported [12]. Consequently, in studies of new animal models or other body organs than presented herein, a test perfusion would be advised.

Finally, we also report that gold-standard immunohistology staining was well feasible after μCT. Thus, the examined tissues could be analyzed further for potential signs of pathology, such as inflammatory processes or fibrotic formation (Fig 5).

Taking together, we show evidence that the presented methodological developments, including the perfusion protocol, the optimized image acquisition and image analysis, provide an improvement over the previously described methods of vascular μCT. They will greatly advance research of microvascular beds in multi-organ diseases, particularly by enabling the reliable quantitative analysis of the vascular fingerprints.

## Supporting information

S1 Table μCT acquisition parameters used for evaluation of the perfusion protocol. (PDF)

S2 Table Steps and Parameters of the image analysis protocols. (PDF)

S3 Table Image acquisition parameters for optimization of the X-ray source power. (Parameters written in grey were the same for all three protocols).
(PDF)

S4 Table Immunostaining protocol after Microfil-perfusion and μCT imaging for several common cardiac antibodies. (PDF)

S1 Fig μCT images were used to evaluate the multi-organ perfusion protocol. Perfusion of the brains, kidneys and tongues appeared most consistent, while the hearts differ to a certain extent in regard to the chamber filling. In the eyes, iris arteries in one animal (#19062) were filled with the contrast agent to a lesser extent. (Image display settings are the same for all the samples of a given organ type).
(PDF)

S2 Fig μCT image of a rat tongue acquired with the “highest resolution”protocol. **A.** An overview of the organ. **B.** Vascular network of the organ. **C.** Visualisation of the tongue surface. The yellow box is the area enlarged in D. **D.** A zoom-in onto the organ surface revealing lingual papillae.
(PDF)

S3 Fig Total organ volumes computed from μCT images acquired with three different protocols. The bars represent group means ± sds (n = 4).
(PDF)

S1 Video Examplar vascular system of the brain (#19063). (MP4)

S2 Video Examplar vascular system of the eye (#19063). (MP4)

S3 Video Example vascular system of the tongue (#19063). (MP4)

S4 Video Example vascular system of the heart (#19063). (MP4)

S5 Video Example vascular system of the kidney (#19063). (MP4)
