# Supplementary material for: μCT imaging of a multi-organ vascular fingerprint in rats
Source: PLoS One. 2024 Oct 14;19(10):e0308601. doi: 10.1371/journal.pone.0308601 (PMC11472947; doi:10.1371/journal.pone.0308601)
Supplement: S1 Table — (PDF) [file pone.0308601.s001.pdf]

µCT imaging of a multi-organ vascular fingerprint in rats

# – Supporting information

Methods

S1 Table. µCT acquisition parameters used for evaluation of the perfusion protocol.

Parameter	Heart	Brain	Kidney	Tongue	Eye
Source voltage (kV)	75	90	90	75	75
Filter	Al 1mm	Al+Cu	Al+Cu	Al 1 mm	Al 1 mm
Exposure time (ms)	820	785	785	820	820
Rotation step (°)	0.2	0.2	0.2	0.2	0.2
Frame average	3	3	3	4	5
Pixel size (µm)	6	6	6	5	4
