# Supplementary material for: μCT imaging of a multi-organ vascular fingerprint in rats
Source: PLoS One. 2024 Oct 14;19(10):e0308601. doi: 10.1371/journal.pone.0308601 (PMC11472947; doi:10.1371/journal.pone.0308601)
Supplement: S2 Table — (PDF) [file pone.0308601.s002.pdf]

μCT imaging of a multi-organ vascular fingerprint in rats

# – Supporting information

Methods

S2 Table. Steps and Parameters of the image analysis protocols.

Heart	Brain	Kidney	Tongue	Eye
Loading image	Loading image	Loading image	Loading image	Loading image (dry sample)
Thresholding: global threshold = 35	Thresholding: global threshold = 28	Thresholding: global threshold = 36	Thresholding: global threshold = 28	Thresholding: global threshold = 30
Morphological operation: opening, r = 2 (3D)	Despeckle: sweep all except the largest object (3D)	Despeckle: sweep all except the largest object (3D)	Despeckle: sweep all except the largest object (3D)	Morphological operation: opening, r = 1 (3D)
Despeckle: sweep all except the largest object (3D)	Despeckle: remove black speckles < 1000 px (3D)	Despeckle: remove black speckles < 1000 px (3D)	Despeckle: remove black speckles < 1000 px (3D)	Despeckle: sweep all except the largest object (3D)
Morphological operation: closing, r = 1 (3D)	Morphological operation: closing, r = 2 (3D)	Morphological operation: closing, r = 1 (3D)	Morphological operation: closing, r = 1 (3D)	Despeckle: remove pores (3D)
Despeckle: remove pores (3D)	Save bitmap	Save bitmap	Save bitmap	Morphological operation: closing, r = 1 (3D)
Save bitmap	3D analysis with basic parameters	3D analysis with basic parameters	3D analysis with basic parameters	Save bitmap
3D analysis with basic parameters	Reloading the image	Reloading the image	Reloading the image	3D Analysis with basic parameters
Reloading the image	Filtering: median, r = 1 (3D)	Filtering: median, r = 1 (3D)	Filtering: median, r = 1 (3D)	Loading the image of the wet sample
Filtering: conditional mean, threshold = 20, r = 3 (3D)	Thresholding: global threshold =50	Thresholding: adaptive median, lower threshold = 64, upper threshold = 87, r = 2 (3D)	Thresholding: global threshold = 54	Filtering: median, r = 1 (3D)
Thresholding: adaptive median, lower threshold = 82, upper threshold = 115, r = 2	Despeckle: remove white speckles < 30 px (3D)	Despeckle: remove white speckles < 30 px (3D)	Despeckle: remove white speckles < 30 px (3D)	Thresholding: adaptive median, lower threshold = 50, upper threshold = 68, r = 2 (3D)

Morphological operation: opening, r = 1 (3D)	Save bitmap	Morphological operation: closing, r = 1 (3D)	Save bitmap	Despeckle: remove white speckles < 30 px (3D)
Despeckle: remove white speckles < 50 px (3D)	3D analysis with basic and additional parameters: structural thickness	Save bitmap	3D analysis with basic and additional parameters: structural thickness	Morphological operation: opening, r = 1 (3D)
Save bitmap		3D analysis with basic and additional parameters: structural thickness		Despeckle: remove white speckles < 30 px (3D)
3D analysis with basic and additional parameters: structural thickness				Morphological operation: closing, r = 1 (3D)
				Save bitmap
				3D analysis with basic and additional parameters: structural thickness
