# Supplementary material for: μCT imaging of a multi-organ vascular fingerprint in rats
Source: PLoS One. 2024 Oct 14;19(10):e0308601. doi: 10.1371/journal.pone.0308601 (PMC11472947; doi:10.1371/journal.pone.0308601)
Supplement: S3 Table — (Parameters written in grey were the same for all three protocols). (PDF) [file pone.0308601.s003.pdf]

µCT imaging of a multi-organ vascular fingerprint in rats

# – Supporting information

Methods

S3 Table. Image acquisition parameters for optimization of the X-ray source power. (Parameters written in grey were the same for all three protocols).

	Highest Resolution				
	Heart	Kidney	Eye	Brain	Tongue
Pixel Size (Px)	5	5	5	5	5
Rotation Step (°)	0.2	0.2	0.2	0.2	0.2
Frame Averaging	2	2	3	2	2
Filter	Cu 0.25mm	Cu 0.25mm	Al 1mm	Al+Cu	Al 1mm
Voltage (kV)	100	100	75	90	75
Number of FOVs	2	2	1	3	2
Current (µA)	40	40	54	45	54
Power (W)	4	4	4	4	4
Exposure Time (ms)	6000	5000	3000	3400	3000
Scan duration	6h:57min	5h:57min	5h:45min	4h:22min	3h:57min
Total Time	13h:54min	11h:54min	5h:45min	13h:6min	7h:54min

	Intermediate Parameters				
	Heart	Kidney	Eye	Brain	Tongue
Current (µA)	80	80	105	90	105
Power (W)	8	8	8	8	8
Exposure Time (ms)	3400	3000	1400	2000	1500
Scan duration	4h:21min	3h:56min	3h:20min	2h:56min	2h:26min
Total Time	8h:42min	7h:52min	3h:20min	8h:48min	4h52min

	Fastest Scanning				
	Heart	Kidney	Eye	Brain	Tongue
Current (µA)	200	200	200	200	200
Power (W)	20	20	15	18	15
Exposure Time (ms)	1250	950	770	725	770
Scan duration	2:11min	1h:53min	2h:25min	1h:42min	1h:42min
Total Time	4h:22min	3h:54min	2h:25min	5h:6min	3h:24min
