# Supplementary material for: μCT imaging of a multi-organ vascular fingerprint in rats
Source: PLoS One. 2024 Oct 14;19(10):e0308601. doi: 10.1371/journal.pone.0308601 (PMC11472947; doi:10.1371/journal.pone.0308601)
Supplement: S4 Table — (PDF) [file pone.0308601.s004.pdf]

µCT imaging of a multi-organ vascular fingerprint in rats

# – Supporting information

Methods

S4 Table. Immunostaining protocol after Microfil-perfusion and µCT imaging for several common cardiac antibodies.

	WGA	CD68	Fibronectin	Collagen Type 1
**Unmasking**	20 min citrate buffer, room temperature (RT)			
**Blocking**	15 min 3% H_2O_2, RT			
**Washing**	3x 5 min dH_2O, RT			
**Blocking**	60 min 10% normal donkey serum (NDS), RT, wet chamber			
**1st antibody**	overnight, WGA, 1:100 (in 10% NDS), 4°C, wet chamber	overnight, anti-ED1, 1:100 (in 10% NDS), 4°C, wet chamber	overnight, anti-Fibronectin, 1:75 (in 10% NDS), 4°C, wet chamber	overnight, anti-Type I Collagen, 1:20 (in 10% NDS), 4°C, wet chamber
**Washing**	3x 5 min 1x phosphate buffer solution (PBS), RT			
**2nd antibody**	none	120 min Cy3-conj. donkey anti-mouse IgG, 1:300 (in 1x PBS), RT, wet chamber	120 min Cy3-conj. donkey anti-rabbit IgG, 1:300 (in 1x PBS), RT, wet chamber	120 min Cy3-conj. donkey anti-goat IgG, 1:300 (in 1x PBS), RT, wet chamber
**Washing**	3x 5 min 1x PBS, RT			
**Covering**	Vectashield / DAPI			
