# Supplementary material for: μCT imaging of a multi-organ vascular fingerprint in rats
Source: PLoS One. 2024 Oct 14;19(10):e0308601. doi: 10.1371/journal.pone.0308601 (PMC11472947; doi:10.1371/journal.pone.0308601)
Supplement: S1 Fig — Perfusion of the brains, kidneys and tongues appeared most consistent, while the hearts differ to a certain extent in regard to the chamber filling. In the eyes, iris arteries in one animal (#19062) were filled with the contrast agent to a lesser extent. (Image display settings are the same for all the samples of a given organ type). (PDF) [file pone.0308601.s005.pdf]

µCT imaging of a multi-organ vascular fingerprint in rats – Supporting information

Results

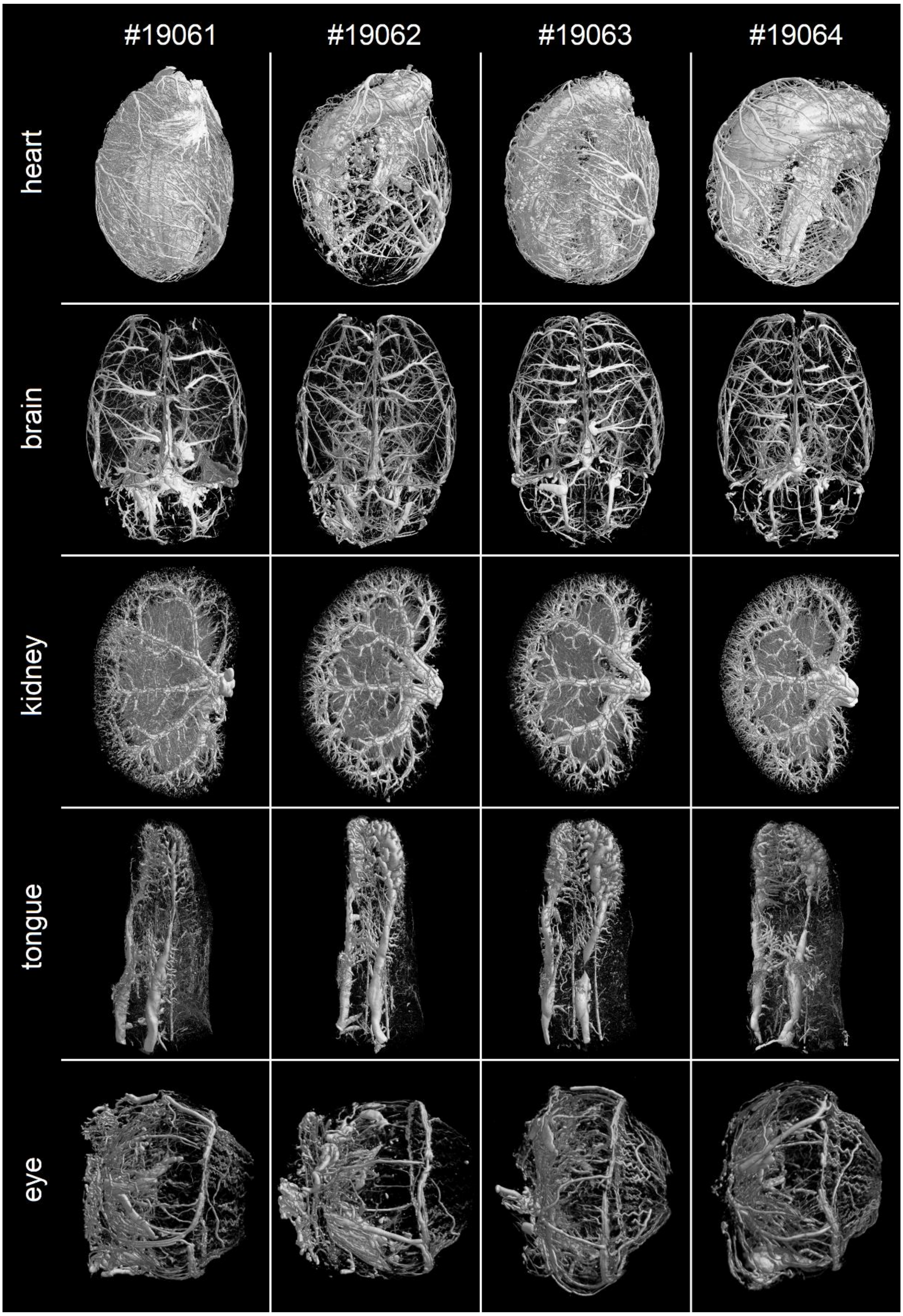

S1 Figure. µCT images were used to evaluate the multi-organ perfusion protocol. Perfusion of the brains, kidneys and tongues appeared most consistent, while the hearts differ to a certain extent in regard to the chamber filling. In the eyes, iris arteries in one animal (#19062) were filled with the contrast agent to a lesser extent. (Image display settings are the same for all the samples of a given organ type).
