# Supplementary material for: μCT imaging of a multi-organ vascular fingerprint in rats
Source: PLoS One. 2024 Oct 14;19(10):e0308601. doi: 10.1371/journal.pone.0308601 (PMC11472947; doi:10.1371/journal.pone.0308601)
Supplement: S2 Fig — A. An overview of the organ. B. Vascular network of the organ. C. Visualisation of the tongue surface. The yellow box is the area enlarged in D. D. A zoom-in onto the organ surface revealing lingual papillae. (PDF) [file pone.0308601.s006.pdf]

µCT imaging of a multi-organ vascular fingerprint in rats

# – Supporting information

Results

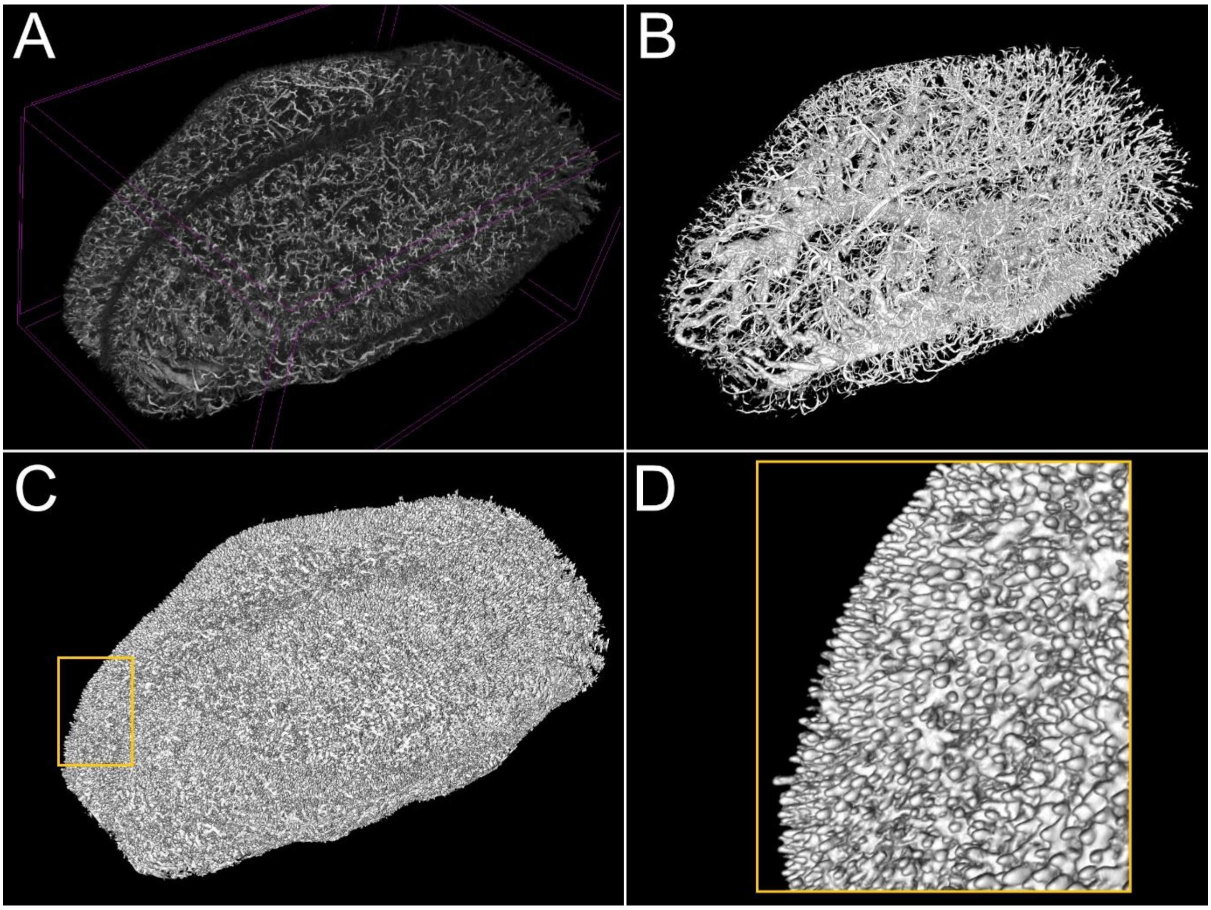

S2 Figure. µCT image of a rat tongue acquired with the "highest resolution" protocol. **A.** An overview of the organ. **B.** Vascular network of the organ. **C.** Visualisation of the tongue surface. The yellow box is the area enlarged in D. **D.** A zoom-in onto the organ surface revealing lingual papillae.
