# Supplementary material for: μCT imaging of a multi-organ vascular fingerprint in rats
Source: PLoS One. 2024 Oct 14;19(10):e0308601. doi: 10.1371/journal.pone.0308601 (PMC11472947; doi:10.1371/journal.pone.0308601)
Supplement: S3 Fig — The bars represent group means ± sds (n = 4). (PDF) [file pone.0308601.s007.pdf]

µCT imaging of a multi-organ vascular fingerprint in rats – Supporting information

Results

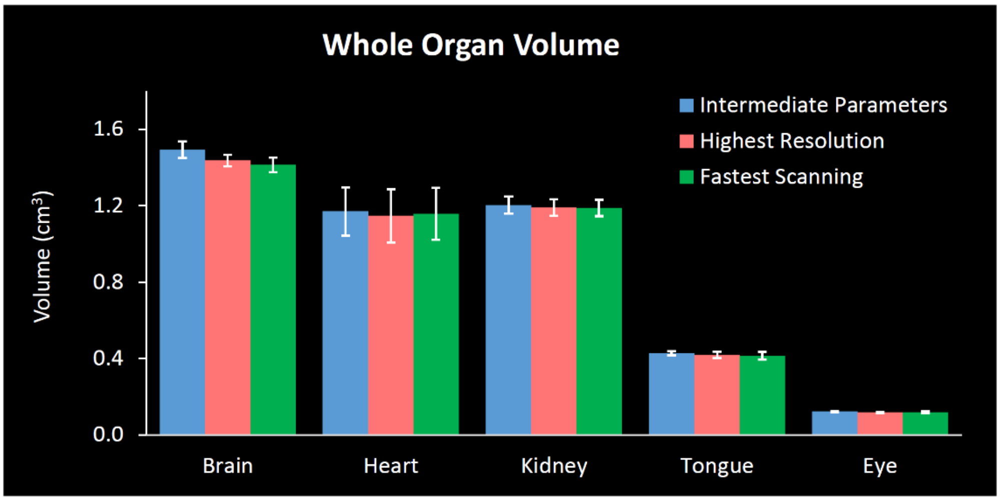

S3 Figure. Total organ volumes computed from µCT images acquired with three different protocols. The bars represent group means ± sds (n = 4).
